# Country and Gender-Specific Achievement of Healthy Nutrition and Physical Activity Guidelines: Latent Class Analysis of 6266 University Students in Egypt, Libya, and Palestine

**DOI:** 10.3390/nu9070738

**Published:** 2017-07-11

**Authors:** Walid El Ansari, Gabriele Berg-Beckhoff

**Affiliations:** 1 Department of Surgery, Hamad General Hospital, Doha 3050, Qatar; 2 College of Medicine, Qatar University, Doha 3050, Qatar; 3 School of Health and Education, University of Skövde, Skövde 541 45, Sweden; 4 Faculty of Applied Sciences, University of Gloucestershire, Gloucester GL53 7TH, UK; 5 Unit for Health Promotion Research, Institute of Public Health, University of Southern Denmark, Niels Bohrs Vej 9, 6700 Esbjerg, Denmark; gbergbeckhoff@health.sdu.dk

**Keywords:** university students, healthy nutrition, physical activity, health promotion, latent class analysis, prevention research, Eastern Mediterranean

## Abstract

Research on healthy behaviour such as physical activity and healthy nutrition and their combination is lacking among university students in Arab countries. The current survey assessed healthy nutrition, and moderate/vigorous physical activity (PA) of 6266 students in Egypt, Libya, and Palestine. We computed a nutrition guideline achievement index using WHO recommendation, as well as the achievement of PA recommendations using guidelines for adults of the American Heart Association guidelines. Latent class regression analysis identified homogenous groups of male and female students, based on their achievements of both guidelines. We examined associations between group membership and achievement of guidelines. A three-class solution model best fitted the data, generating three student Groups: “Healthy Eaters” (7.7% of females, 10.8% of males), “Physically Active” (21.7% of females, 25.8% of males), and “Low Healthy Behaviour” (70.6% of females, 63.4% of males). We did not observe a latent class that exhibited combined healthy behaviours (physically active and healthy eaters), and there were no major differences between countries. We observed a very low rate of healthy nutrition (≈10% of students achieved greater than four of the eight nutrition guidelines), with little gender differences across the countries. About 18–47% of students achieved the PA guidelines, depending on country and gender, more often among males. Few females achieved the PA guidelines, particularly in Libya and Palestine. Culturally adapted multi-behavioural interventions need to encourage healthy lifestyles, nutrition and PA behaviours. National policies need to promote active living while addressing cultural, geographic, and other barriers to young adults’ engagement in PA.

## 1. Introduction

A recent article debated the dramatic increase of Diabetes across most Arab countries [1], suggesting efforts to combat the spread of this disease. High blood pressure and high blood cholesterol, inadequate fruit/vegetables intake, overweight/obesity, physical inactivity and tobacco use are risk factors of non-communicable diseases including Diabetes [2]. Hence, healthy lifestyles that include healthy nutrition and physical activity (PA) can prevent obesity and Diabetes.

Healthy nutrition is critical for health. Although this is particularly true for young adults [3,4], university students’ life transition often results in poor nutrition and unhealthy weight gain [5], and those with healthier nutrition report better self-reported health and less health complaints [6]. Nevertheless, in Egypt, about half of the medical students were overweight/obese [7], leading to recommendations for nutritional education and physical activity (PA) programs to confront the rising rate of overweight/obesity. Similarly, Libyan adolescents had the highest prevalence of overweight (26.6%) across seven Arab countries [8]. Likewise, in Kuwait, health sciences students had suboptimal diet [9]; and, in Lebanon, the prevalence of overweight/obese students was 32.2%, and their dietary consumption was below the recommended guidelines [10]. In Iran, most students needed to improve their diet (only 28.5% had a good diet) [11]; and, in the United Arab Emirates, students had a high prevalence of consumption of dietary supplements [12]. Low rates of healthy nutrition are evident among university students across the Eastern Mediterranean Region (EMR).

In terms of PA, low PA levels are associated with physical symptoms/illness among university students [13], and with negative health consequences, e.g., chronic heart and respiratory diseases, and unhealthy weight gain [14]. Notwithstanding, in Egypt, about two thirds of the medical students exercised, and 26.9% exercised for <2 h per week [15]. Likewise, in Libya, few students achieved the recommended PA guidelines, where female students were particularly at risk [16]; and sedentary lifestyle was common among Libyan adolescents/young adults [17]. Similarly, in Kuwait, university students had suboptimal PA [9].

The published literature reveals several gaps. Generally, few studies have assessed nutrition in conjunction with PA across the Arab EMR nations, as when such research was undertaken, it was mostly in Western countries, e.g., Poland [18] or Australia [19]. Indeed, Arab country comparisons are very sparse in terms of university students’ nutrition and PA behaviours, despite their importance for these young adults, particularly to prevent type 2 Diabetes in later life. 

Therefore, the current study bridges the knowledge gap on gender-specific healthy nutrition and PA among university students in Egypt, Libya, and Palestine. We identify and describe the congregation of two behavioural risk factors (nutrition behaviour and PA) among these young adults at a range of universities/colleges across the three countries; and define country and gender-specific subgroups that would benefit from health promotion actions. We employed a latent class analysis approach to examine the association between subgroup membership and healthy nutrition and PA [20]. Collectively, these features highlight the importance of the findings of the current study, and its contribution to the evidence base.

## 2. Materials and Methods

### 2.1. Ethics, Study Design, Sample and Procedures

The current student health and wellbeing cross sectional study employed an identical self-administered paper questionnaire administered at several Universities in Egypt, Libya and Palestine—Gaza Strip. All participating institutions across the three countries provided ethical approval for the study. Participation was anonymous, students consented for participation in the study, and all data were confidential and protected. The study did not distribute the self-administered questionnaire to all students at each faculty/institution, rather data were collected usually at the end of lectures where questionnaires were distributed to participants attending selected classes, and then collected after completion. Participation in the survey was voluntary, and to encourage participation and improve the response rates (without coercion), we highlighted to students the importance of their participation. For instance, in Egypt, a 10% sample of students was achieved out of all the students in university, and, in effect, our sample can be considered a convenience sample. 

Response rates were computed based on the number of completed questionnaires that were returned by participants at each institution, compared to the number of questionnaires distributed.

In Egypt, data were collected (2009–2010) from all faculties at the University of Assiut, Assiut, Egypt (Business, Engineering, Education, Arts, Social Work, Sciences, Physical Education, Computers and Information, Veterinary Medicine, Specific Education, Agriculture). This yielded 3271 students; 396 participants were excluded due to missing data on the variables under examination, leaving 1543 females (52.6%) and 1362 males (47.4%). Response rate was ≈90%.

In Libya, 9 institutions (6 universities and 3 colleges) in 7 cities (Misurata, Sabbha, Zawea, Sirt, Al Bida, Benghazi, and Tripoli) participated (2008–2009). Participating students represented Faculties such as Agriculture, Business, Education, Law, Mechanical Engineering, Medical Science, Medical Technology and others. A total of 1567 participants completed the survey; 267 questionnaires were excluded (missing data on the variables under examination) leaving 861 females (66.2%) and 439 males (33.8%). Response rate was 74.6%.

In Palestine (Gaza strip), students at 5 universities and 3 colleges participated in the study during 2013. Private, public and governmental universities were included representing many disciplines (Al-Azhar University, Al-Aqsa University, Palestine University, The Islamic University, Gaza University, College of Applied Sciences, Palestine College of Nursing, Dar Al Dawa, Humanities College). A total of 1428 students completed the survey; 161 were excluded due to missing values on the variables under examination, leaving 743 females (58.6%) and 524 males (41.4%) included the analysis. Response rate was ≈85%.

### 2.2. Research Instruments and Data Collection

The study included self-reported socio-demographic information, and data on physical activity and nutritional habits (consumption frequency of 12 food groups).

*Assessment of food intake*: Students self-reported their food intake in a food frequency questionnaire (FFQ) that measured their usual consumption (How often do you eat the following foods?) of 12 food groups (each food group individually). The response scale comprised: 5 = “several times a day”, 4 = “daily”, 3 = “several times a week”, 2 = “1–4 times a month”, and 1 = “never”. The instrument was based on pre-existing food frequency questionnaires, adapted for the study [21] and analogous to validated FFQs [22,23]. The categories “several times a week” and “daily” were collapsed together for the analysis. 

*Healthy Nutrition guideline* achievement *index*: using students’ FFQ responses, we computed an objective dietary guideline achievement index, with a maximum 8 points (8 guidelines), derived from 8 foods: (1) sweets, cookies and snacks; (2) Fast food/canned food; (3) lemonade/soft drinks; (4) fruits; (5) salad and raw vegetables; (6) cooked vegetables; (7) meat; and (8) fish. We used the WHO dietary guidelines recommendations for Eastern Mediterranean region [24], hence, for the number of daily fruit, raw and cooked vegetables servings, the cut-off was “daily” or “several times a day”; and for meat, the cut-off was “less than daily”; and for fish the cut-off was “several times/week”. Given that sweets, cake/cookies, snacks, fast food/canned food and lemonade/soft drinks should be eaten “only occasionally”, we employed our FFQ’s categories “1–4 times a month” and “never” as recommended. To consider all sweets, cake/cookies and snacks together, we summed up all three foods into “Sweets, cookies and snacks score”, and healthy nutrition was considered present if this score was ≤6, corresponding to three times the intake of these items being “less often than 1–4 times a month”. Each of the fast food/canned food and lemonade/soft drinks were included as individual items in computing the guideline achievement index. Finally, milk and cereals were not included in computing the achievement index as milk consumption is healthy only if lactose intolerance is not present, a condition that is common (74%) among Egyptian children [25]; and the information about cereals is generally too unspecific to be categorized as healthy/unhealthy nutrition.

*Physical activity:* Vigorous PA (VPA) was measured by: “On how many of the past 7 days did you participate in vigorous exercise for at least 20 min?” Participants answered with 0–7 days. We used a cut-off of ≥3 days/week as achievement of the PA guidelines [14]. Moderate PA (MPA) was measured by: (“On how many of the past 7 days did you participate in moderate exercise for at least 30 min?” Participants answered with 0–7 days. We used the American College of Sports Medicine and the American Heart Association cut-off of ≥5 days/week as achievement of PA guidelines [14]. 

Additional variables included: (1) age; (2) gender; (3) respondent’s subjective economic situation (“How sufficient is your income?”, coded into sufficient vs. not sufficient); and (4) living situation/arrangements during university terms (“Where do you live during university/college term time?” coded into living at parental home vs. not living at home). Body mass index (BMI) was calculated from self-reported weight and height (kg/m^2^).

### 2.3. Statistical Analysis

Analysis was conducted in SAS Version 9.4 (SAS Institute Inc., Cary, NC, USA). For descriptive purposes, we present percentages, and chi-square test was used to compare the characteristics and habits between the countries. To explain latent structures in the student’s healthy behaviour with regard to PA and nutrition, we undertook a latent class analysis (PROC LCA). This analysis involved segmenting our population into mutually exclusive latent classes, where each class contains items with similar patterns/properties, but dissimilar to those in different clusters [20]. The idea behind latent class analysis is to construct a latent variable that explains healthy behaviour. This variable cannot be observed directly, but rather, is inferred from multiple observed categorical items. The number of latent classes was chosen according to the smallest values of Bayesian information criterion (BIC) and the Akaike information criterion (AIC). The final latent class was calculated considering all three countries in one model, and was additionally adjusted for the countries.

## 3. Results

Table 1 shows that there were more female students in all three countries. The percentage of overweight students was most pronounced in Egypt (28.9%), followed by Libya (27.9%) and Palestine (24.5%). The self-reported sufficient economic situation exhibited the same gradient, and was highest in Egypt (75.9%), followed by Libya (72.9%) and Palestine (60.7%).

Table 2 presents the daily eating habits. Fresh fruits and salads were eaten less often in Palestine; daily cooked vegetables were consumed most often in Libya. No stringent differences were observed with regard to meat, fish, and cereals consumption. However, daily meat was eaten rarely (<30% of the students ate meat daily), but fish was consumed more often (>50% of the sample ate fish daily). Milk consumption was less common in Libya. Unhealthy eating, e.g., sweet, cakes/cookies, snacks, fast food, and soft drinks was most often in Egypt, followed by Libya (snack and soft drinks were less common) and Palestine, where only fast food was eaten by >40% daily.

Table 3 depicts healthy behaviour in terms of PA and nutrition. Overall PA (measured as VPA and MPA) in males did not differ across countries. PA was less common among females. Only in Egypt females had PA comparable to the male counterparts, where overall VMPA was ≈40%, less but similar to the male PA (47.43%). Far lower prevalence of female student’s PA was observed in Libya and Palestine. Healthy nutrition was rare in all countries, with no gender differences.

Table 4 shows the BIC/AIC values used to estimate the best number of latent classes, suggesting that the best number of latent classes that fit the data was three (minimum BIC and AIC values). Table 5 depicts the latent class analysis undertaken for each country individually (Table 5a), and collectively for the three countries together (Table 5b). In all four models, latent class analysis showed that model that best fitted the data comprised a three-class solution, which we labelled. The latent class estimates item-response probabilities conditional on class membership. The distribution of these probabilities among the different classes is used to calculate the prevalence in each class, which is termed “posterior prevalence”. “Healthy Eaters” had a posterior prevalence of 7.7%**_♀_**, 10.8%**_♂_**; “Physically Active” students had a posterior prevalence of 21.7**_♀_** and 25.8%**_♂_**; and “Students with Low Healthy Behaviour” had posterior prevalence 70.6%**_♀_** and 63.4%**_♂_**. No latent class explained physically active and healthy eaters in any of the countries. Individuals within the Healthy Eaters subgroup had increased probability of healthy diet and fruit consumption. Students within the Physically Active subgroup had a relatively increased probability of moderate PA and vigorous PA. Finally, the Low Healthy Behaviour subgroup members had a decreased probability of all the listed healthy behaviours. The female healthy eaters comprised the smallest percentage among the students (7.7%, Table 5b). The largest group and that which was also evident in all three countries and across both sexes comprised those students with Low Healthy Behaviours (70.6%**_♀_**, 63.4%**_♂_**, Table 5b).

## 4. Discussion

Overall, across the three countries, we observed a very low rate of healthy nutrition, where only ≈10% of the university students surveyed in these countries achieved ≥ 4 out of the eight healthy nutrition recommendations examined. As for latent classes of healthy behaviour groups, we observed no differences between countries and gender, even though there were significant differences between single nutritional behaviours and different physical activity types between countries and gender. Generally, students’ dietary and lifestyle behaviours globally display controversial results. Most research reported a high tendency among the majority of students to engage in unhealthy dietary and lifestyle habits. Our findings support other research that found low fruits and vegetables intake and high fast food consumption in Pakistan [26], Greece [27], and Saudi Arabia [28]. We also agree with Lebanon, where Health Sciences university students’ dietary consumption patterns were below recommended levels [10]; and with the USA, where university students’ dietary consumption patterns, and knowledge of healthy/unhealthy diet habits needed improvement [29].

Generally, across the three countries, there has been progressive erosion of their traditional diets in the recent periods associated with the increased urbanization, and introduction of new foods and eating customs. Such changes in traditional diets are by no means unique to these three countries, and are witnessed widely in the context of the nutrition transition globally. For instance, in Egypt, changes in food consumption patterns are evident since decades, where the typical Egyptian diet (bread, legumes, fresh dark vegetables, other vegetables, and fruits) has gradually been replaced by fast food and highly processed items [30]. In addition, it remains unclear whether the observed low healthy nutrition among our student population was due to lack of nutrition consciousness or lack of knowledge. For instance, among pharmacy students in Egypt, in terms of healthier eating habits and lifestyle, a higher correlation existed between the students’ health manifestations and knowledge (*r* = 0.32) than consciousness (*r* = 0.28) [31]. Likewise, in Libya since the late 1960s to early 2000s, the average calories available per individual per day have increased [32]. A Libyan adult consumes an extra 1183 kcal daily, and the Libyan diet is high in calories and rich in fat, while being low in vegetables and fruits [33,34]. Similarly, food consumption and dietary habits in Palestine and the neighbouring EMR have changed distinctly during the past decades, where high consumption of fat-rich foods and calories and sedentary lifestyles have key roles in the increase in obesity [35,36,37].

A sizeable proportion of our participants were students in the health fields, and whilst it is anticipated that those would have greater knowledge than other students, there is no evidence to indicate that this knowledge is actually translated into healthy dietary/lifestyle practices [26]. Hence, views about what constitutes a healthy diet are important, where Chinese and American undergraduate students’ perspectives on healthy eating indicated that understanding the views about what constitutes a healthy diet may inform design of culturally tailored behaviour change interventions [38]. In agreement, significant differences in dietary patterns were reported between Arabs and Jews in Israel, suggesting a need for culturally congruent dietary interventions to address nutrition-related chronic disease disparities [39]. Individuals translate healthy eating in complex and diverse ways that reflect their personal, social, and cultural experiences, as well as their environments. Their meanings include, but are broader than, the researchers’ views about food composition and health outcomes. Identity (self-concept), social settings, resources, food availability, and other conflicting considerations may explain why people may not be consuming according to their healthy eating ideals [40]. Hence, across different countries, cultures and environments, such diverse views may reflect food-related messages to which individuals are exposed through the media and through the educational systems in their respective countries [38]. Future research would benefit from analyses of the relationships between nutritional knowledge, nutrition consciousness, views about what constitutes a healthy diet on the one hand; and the media and how these interrelated factors collectively interact and influence un/healthy nutritional habits in different countries. Nevertheless, the low rates of healthy nutrition habits we observed are a concern due to their long-term contributions to obesity, diabetes, hypertension and other conditions.

As for gender, we found little gender differences in the very low rate of healthy nutrition across the three countries. However, examining our latent classes, the “Healthy Eaters” latent class was more pronounced in males (22.7%) compared to females (6.0%). The literature on such gender differences is inconsistent and not well understood. On the one hand, in the USA, female college students exhibited greater nutrition knowledge than males (women mean nutrition score was 5 points higher than men, P = 0.01) [41]; and in Lebanon, male students had a higher consumption of westernized dietary pattern as compared to female students who reported a higher vegetarian diet [42]. In contrast, others [43,44] found that males consumed, compared to females, fast food and meat products more often, and fruits and vegetables less often. Such patterns highlight the interrelated aspects of attitudes and affect, nutrition knowledge, and intentions and willingness to eat healthy and unhealthy foods [45]. Females are more concerned about their body weight than males [29,36]; or may have higher health awareness [46], enhanced nutrition knowledge [47], and better understanding about what comprises a “healthy diet” [29]. Conversely, in the USA, female college students were disposed to unhealthy eating patterns. Future research can test whether cultural differences can explain the incongruent gender differences of healthy nutrition between Western and Arab countries.

In terms of PA, we observed that the achieved recommendation for vigorous and moderate PA ranged from 18–47% depending on country and gender, and that the PA recommendation was achieved more often among males than females. Our findings support similar research: in Egypt [48] and in Kuwait [49] 33.8% and 45% of the surveyed students respectively were physically inactive; in Libya, 34.7% of students practised regular PA [50]; and across the Mediterranean region, the prevalence of physical inactivity was 39% [51]. Among adolescents in seven Arab countries, the main barriers for PA were lack of motivation to do PA, less support from teachers, and lack of time [8].

Our latent class analyses found only three homogenous mutually exclusive groups: “Healthy Eaters”, “Physically Active” and a third group characterised by “Low Healthy Behaviour”. We did not detect a latent class that exhibited several simultaneous healthy behaviours. This finding contrasts with other latent class analyses in Western countries, where healthy/unhealthy behaviours were grouped together. For instance, in the USA, Laska [52] reported four different classes in a group of College Youth aged 18–25 of years. There were three similar groups in both genders; which they entitled “poor lifestyle” (40.0%**_♀_**, 9.2%**_♂_**), “higher risk” (24.3%**_♀_**, 33.6%**_♂_**), “moderate life style, low risk” (40.0%**_♀_** 51.0%**_♂_**). Furthermore, for both genders, one additional fourth group was found and was given different titles by gender: in women, it was called “health conscious” (15.4%**_♀_**); and in men, “classic jocks” (6.2%**_♂_**). Future research would benefit from considering cultural differences to help explain such discrepancies in health behaviours between Western and Arab countries.

Young adulthood is generally characterized by a steep decline in PA levels [53], and we observed that there was a relatively higher prevalence of PA among females in Egypt compared to Libya and Palestine. This might be due to the relatively more liberal views on females and social norms and roles in Egypt, where generally, Egypt has traditionally been known to be a more “liberally open” country compared to Libya and Palestine. Such more liberal views on gender might be a reason that females exercising, jogging or walking alone in Egypt could be more freely acceptable, as there exists relatively more tolerance to such activities than in neighbouring countries. However, conversely, others have proposed that for Saudi university students, it was the lack of facilities and lack of encouragement, rather than the lack of knowledge and/or restrictions from families/society, that seem to hamper female students’ PA [54].

One new finding from our latent class analysis is that all the models showed that the three-class model had the best fit. This implicitly means that in none of these countries there seemed to be a higher probability to combine healthy nutrition with PA. This finding has important implications. It is essential to recommend, explain, and introduce healthy nutrition in fitness centres, gyms and other places where PA is undertaken. At these places, it might be easier to motivate persons to eat healthy, as they are already interested in healthy lifestyle with regard to PA. However, as our results indicate, most students are still not achieving the healthy nutrition recommendations.

An important point is the magnitude (size) of the “Low Healthy Behaviours” group (neither healthy nutrition nor PA). About 50% of the students in Egypt, Libya and Palestine neither achieved the healthy nutrition recommendation nor the PA recommendation. This population is particularly at risk of type 2 Diabetes in later life, and could contribute to the dramatic increase in Diabetes across the Arab countries [1]. Therefore, promotion of healthy behaviours needs to be introduced at many institutions, e.g., at universities. Facilities for female PA need to be constructed, and the importance of healthy nutrition and PA need to be promoted at all universities. 

This study has limitations. As with other studies, the majority of nutrition and PA assessment methods commonly used in research are subject to recall and social desirability biases, which might result in over- or under-reporting of behaviours. We did not objectively measure food consumption, and participants’ self-reported dietary food consumption may be subject to sociability bias. Future research should consider such limitations. For some food groups, we did not assess serving sizes, which might hamper the calculation of a precisely correct healthy eating guideline achievement index. The FFQ was not compared against objective methods of food consumption measurement. Nonetheless, the tool was comparable to other published FFQs that have been validated [22,23]. Our sample remains short of being representative of all higher education students in each country, as with most other published health and wellbeing surveys [55,56]. Hence, generalizability of any findings needs to be cautious. Despite these limitations, the study has strengths. Our large sample comprised students from three countries and several universities and faculties and many scientific disciplines. Females were overrepresented (a reality at higher education institutions across the globe), hence we analysed the relationships for the whole sample and by gender to avoid potential confounding gender effects.

## 5. Conclusions

There is a lack of healthy behaviours among university students in Egypt, Libya and Palestine. Particularly, with the rising Diabetes rates in the Arab world, more effort is necessary to promote healthy eating and PA in these countries. The development of nutritional education and PA programs could tackle the problem of increasing overweight and obese university students. Tailored efforts should introduce healthy behaviours among university students. Future research could examine the most effective intervention strategies. Attention is also required to the national policies that promote active living, along with addressing the cultural, geographic, and other barriers to engagement of young adult males and females in PA.

## Figures and Tables

**Table 1:** Characteristics of the sample across three countries.

	Egypt		Libya		Palestine		*p* *
	N	%	N	%	N	%	
**Sex**							
Female	1543	52.63	861	66.23	743	58.64	<0.0001
Male	1362	47.37	439	33.77	524	41.36	
**Age group** (years)							
16–18	1247	43.86	139	10.69	154	12.23	<0.0001
19–21	1485	52.23	752	57.85	778	61.80	
22–30	111	3.90	409	31.46	327	25.97	
**BMI** (kg/m^2^)							
Below normal (≤18)	114	4.05	62	5.82	69	6.12	0.003
Normal (18–25)	1888	67.02	707	66.32	783	69.41	
Overweight (>25)	815	28.93	297	27.86	276	24.47	
**Economic situation**							
Always/mostly sufficient	2181	75.86	948	72.92	769	60.69	<0.0001
Always/mostly insufficient	694	24.14	352	27.08	498	39.31	

* Chi-square test comparing countries.

**Table 2:** Prevalence of food intake (FFQ).

	Egypt		Libya		Palestine		*p* *	
	Female	Male	Female	Male	Female	Male	Female	Male
**N**	1513	1362	861	439	743	524		
% of students eating daily								
Unhealthy nutrition								
Sweets	37.67	51.84	18.47	30.98	14.80	16.79	<0.0001	<0.0001
Cake/cookies	39.19	51.03	49.83	57.86	22.61	19.66	<0.0001	<0.0001
Snacks	26.97	41.92	16.96	25.97	16.55	20.23	<0.0001	<0.0001
Fast food, canned food	48.91	33.26	53.54	53.30	46.30	41.03	0.02	<0.0001
Lemonade, soft drinks	43.42	42.80	17.89	23.23	34.32	25.19	<0.0001	<0.0001
Healthy nutrition								
Fresh fruits	29.35	33.48	31.24	31.66	22.88	19.27	0.0005	<0.0001
Salad, raw vegetables	47.52	32.89	34.84	38.27	22.61	23.09	<0.0001	<0.0001
Cooked vegetables	17.38	20.93	43.21	50.57	34.86	29.77	<0.0001	<0.0001
Other nutritional aspects								
Meat	13.62	20.04	19.40	17.54	29.48	20.80	<0.0001	0.41
Fish, sea food	67.94	66.23	72.24	66.74	55.05	47.14	<0.0001	<0.0001
Milk, milk products	57.51	52.86	21.02	25.74	47.69	41.20	<0.0001	<0.0001
Cereals, their products	14.58	12.64	19.28	15.03	19.03	14.94	0.003	0.27

FFQ: food frequency questionnaire; * Chi-square test comparing countries.

**Table 3:** Prevalence of healthy nutrition and physical activity among students in Egypt, Libya and Palestine.

	Egypt		Libya		Palestine		*p* *
	N	%	N	%	N	%	
**Female**							
**Physical activity**							
Vigorous PA ^£^	279	18.44	131	15.21	130	17.50	0.14
Moderate PA ^$^	446	29.48	50	5.81	104	14.00	<0.0001
Vigorous or moderate PA ^#^	596	39.39	154	17.89	195	26.24	<0.0001
**Healthy nutrition**							
≥4 recommendations ^¶^	122	8.06	98	11.38	83	11.17	0.01
High fruit & vegetables consumption **	270	17.85	206	23.93	98	13.19	<0.0001
**Male**							
**Physical activity**							
Vigorous PA ^£^	420	30.84	166	37.81	194	37.02	0.004
Moderate PA ^$^	415	30.47	80	18.22	104	19.85	<0.0001
Vigorous or moderate PA ^#^	646	47.43	184	41.91	212	40.46	0.01
**Healthy nutrition**							
≥4 recommendations ^¶^	67	4.92	53	12.07	72	13.74	<0.0001
High fruit & vegetable consumption **	207	15.20	111	25.28	65	12.40	<0.0001

PA: physical activity; ^£^ Vigorous PA: ≥3 days/week in vigorous exercise for at least 20 min; ^$^ Moderate PA: ≥5 days/week in moderate exercise for at least 30 min; ^#^ Vigorous or moderate PA: either vigorous or moderate PA; ^¶^ follow ≥4 healthy nutrition recommendation described in method section; ** Fruit and vegetable score ≥6; * Chi-square test comparing countries.

**Table 4:** BIC and AIC values used to estimate the best number of latent classes.

			Number of Latent Classes			
		**N**	**2**	**3**	**4**	**5**
**Egypt**	BIC	2875	259.97	**230.09**	305.78	384.51
	AIC	2875	152.62	**63.10**	79.16	98.24
**Libya**	BIC	1300	210.53	**208.18**	275.25	344.87
	AIC	1300	117.46	**63.41**	78.79	96.70
**Gaza**	BIC	1267	281.19	**213.28**	272.44	343.80
	AIC	1267	188.59	**69.23**	76.96	96.87
**Overall**	BIC	5442	370.29	**262.16**	338.08	412.99
	AIC	5442	251.45	**77.24**	87.21	96.10

BIC: Bayesian information criterion; AIC: Akaike information criterion; bolded cells show the minimum BIC and AIC values.

**Table:** (**a**)

		Egypt			Libya		Palestine		
	Latent class								
	Healthy Eaters	Physically Active	Low Healthy Behaviour	Healthy Eaters	Physically Active	Low Healthy Behaviour	Healthy Eaters	Physically Active	Low Healthy Behaviour
**Female** (% in each latent class *)	10.9	41.3	47.8	16.6	9.1	74.3	3.5	34.3	62.2
Healthy diet	**0.71**	0.00	0.00	0.47	0.12	0.03	0.34	0.04	0.14
Fruit consumption	**0.56**	0.09	0.16	**0.68**	0.23	0.14	**0.67**	0.01	0.17
Moderate PA	0.33	0.49	0.12	0.05	0.43	0.01	**0.74**	0.32	0.00
Vigorous PA	0.21	0.37	0.01	0.02	**0.75**	0.11	0.36	0.37	0.06
**Male** (% in each latent class *)	17.6	20.7	61.7	22.2	29.4	48.4	33.1	30.5	36.4
Healthy diet	0.21	0.05	0.00	0.30	0.14	0.03	0.29	0.12	0.00
Fruit consumption	**0.68**	0.08	0.02	**0.85**	0.19	0.01	0.31	0.07	0.00
Moderate PA	0.20	**0.58**	0.24	0.01	**0.57**	0.02	0.01	**0.53**	0.09
Vigorous PA	0.14	**0.93**	0.15	0.04	**0.81**	0.26	0.05	**0.97**	0.15

**Table:** (**b**)

	Three Countries Together		
	Latent class		
	Healthy Eaters	Physically Active	Low Healthy Behaviour
**Female** (% in each latent class *)	7.7	21.7	70.6
Healthy diet	0.48	0.05	0.07
Fruit consumption	**0.98**	0.10	0.12
Moderate PA	0.20	**0.86**	0.00
Vigorous PA	0.12	0.33	0.13
**Male** (% in each latent class *)	10.8	25.8	63.4
Healthy diet	0.30	0.05	0.06
Fruit consumption	**0.98**	0.09	0.05
Moderate PA	0.10	**0.94**	0.00
Vigorous PA	0.13	**0.58**	0.27

* Per cent in each latent class is based on the given posterior probability prevalence; each single estimate for each healthy behaviour explains the probability for the response category; bolded cells indicate item response probability within each class of ≥0.50.
